# POU4F2/Brn-3b transcription factor is associated with survival and drug resistance in human ovarian cancer cells

**DOI:** 10.18632/oncotarget.26371

**Published:** 2018-12-04

**Authors:** Lauren J. Maskell, Anupam V. Mahadeo, Vishwanie S. Budhram-Mahadeo

**Affiliations:** ^1^ Molecular Biology Development and Disease, University College London, London, UK; ^2^ Stony Brook University, Stony Brook, NY, USA

**Keywords:** POU4F2/Brn-3b, ovarian cancer, siRNA, drug resistance

## Abstract

The development of drug resistance following treatment with chemotherapeutic agents such as cisplatin (cis) and paclitaxel (pax) contributes to high morbidity and mortality in ovarian cancers. However, the molecular mechanisms underlying such changes are not well understood. In this study, we demonstrate that the Brn-3b transcription factor was increased in different ovarian cancer cells including SKOV3 and A2780 following treatment with cis and pax. Furthermore, sustained increases in Brn-3b were associated with survival in drug resistant cells and correlated with elevated HSP27 expression. In contrast, targeting Brn-3b for reduction using short interfering RNA (siRNA) also resulted in attenuated HSP27 expression. Importantly, blocking Brn-3b expression with siRNA in SKOV3 cells was associated with reduced cell numbers at baseline but also increased cell death after further treatment, indicating sensitization of cells. Similar results were obtained in the metastatic IP1 cell line derived from ascites of mice bearing SKOV3 tumours. These findings suggest that increased Brn-3b may confer resistance to chemotherapeutic drugs in ovarian cancer cells by regulating key target genes such as HSP27 and that targeting Brn-3b may provide a novel mechanism for treatment of drug resistant ovarian cancers.

## INTRODUCTION

Ovarian cancers are the seventh most common cancers in women worldwide but have high mortality, with 5 year survival of 30–50% [1]. Such high mortality arises in part due to the silent nature of this disease since many symptoms are shared with other common conditions [2]. Consequently only ∼15–20% of ovarian cancers are diagnosed at stage I, when the disease is limited to the ovaries and responds effectively to treatments such as surgery. In the majority of cases, patients present with more advanced disease that has spread beyond the ovary to involve the pelvic organs (stage II), abdomen (stage III) or beyond the peritoneal cavity (stage IV), which have poor prognosis and reduced survival rates [3, 4].

Such advanced diseases are commonly treated with a combination of surgery and chemotherapeutic drugs such as cisplatin and paclitaxel, which have been established as first line treatment of such cancers [5, 6]. However, drug resistance remains a major problem particularly in the higher stage cancers because despite initial responsiveness to treatment, the majority of patients eventually relapse with drug-resistant cancer [7–9] which accounts for >90% mortality in patients with metastatic diseases.

The molecular basis by which ovarian cancer cells acquire drug resistance and metastatic potential are not fully understood but such complex processes are highly dependent on changes in cellular genes that enhance survival and confer migratory potential. In this regard, transcription factors which regulate the expression of multiple, tissue-specific target genes will be important for driving such events in cancer cells. Brn-3b is a POU (Pit-Oct-Unc) homeodomain transcription factor, which has been implicated in regulating diverse tumorigenic processes in breast cancer and childhood neuroblastomas. For instance, Brn-3b overexpression enhances cell proliferation *in vitro* and tumour growth *in vivo* [10] while reducing Brn-3b is sufficient to inhibit proliferation and slow tumour growth [11]. However, Brn-3b is also induced following treatment with chemotherapeutic drugs such as cisplatin and high levels also confer drug resistance and increased migratory potential [11, 12]. In line with this, Brn-3b protein is elevated in >60% of breast cancers and >70% of childhood neuroblastomas [13, 14].

As a transcription factor, Brn-3b mediates such diverse effects by complex regulation of multiple target genes. For example, the growth promoting effects of Brn-3b are associated with transactivation of cell cycle proteins cyclinD1/CDK4 [14, 15] and repression of the tumour suppressor gene *BRCA1* [13], which inhibits the cell cycle or activates apoptosis in breast cancer cells. In contrast, when Brn-3b is increased in response to drug treatment, it regulates distinct subsets of genes that can cause different cellular responses. For instance, Brn-3b represses the expression of the adhesion molecule γ-catenin (plakoglobin) [16] which normally represses growth and migration of cancer cells [17] but strongly activates the small heat-shock protein, HSP27 which increases migration in cancer cells but also confers protection from apoptosis [18]. In fact, cooperation between Brn-3b and the oestrogen receptor (ER) is required for maximal stimulation of HSP27 in breast cancer cells suggesting that this transcription factor is important in driving HSP27 expression. HSP27 has been implicated in metastatic ovarian cancers and is considered as a predictor of poor survival in patients with ovarian tumours [19, 20]. Furthermore, reducing HSP27 in ovarian cancer cells confers increased sensitivity to drugs such as paclitaxel suggesting that increased expression of this heat-shock protein will be relevant for conferring drug resistance [21].

In this study, we demonstrated that Brn-3b protein expression was increased in human ovarian cancer cell lines such as SKOV3 and A2780 following treatment with chemotherapeutic drugs such as cisplatin and paclitaxel, which are commonly used for treatment of ovarian cancers. Sustained increases in Brn-3b protein was detected in drug-resistant SKOV3 cells either induced by chronic drug treatment (>2 weeks) or in established SKOV3-IP1 cells. In drug resistant cells, elevated Brn-3b levels correlated with expression of its known target gene, HSP27, whereas blocking Brn-3b using short interfering RNA (siRNA) resulted in loss of HSP27 expression. Furthermore, Brn-3b siRNA reduced cell viability at baseline but also sensitised cells to drug treatment. These results and potential implications for controlling the growth of ovarian cancer cells and responses to chemotherapeutic treatment are discussed here.

## RESULTS

### Induction of POU4F2/Brn-3b in ovarian cancer cells by cisplatin

**Figure 1:**
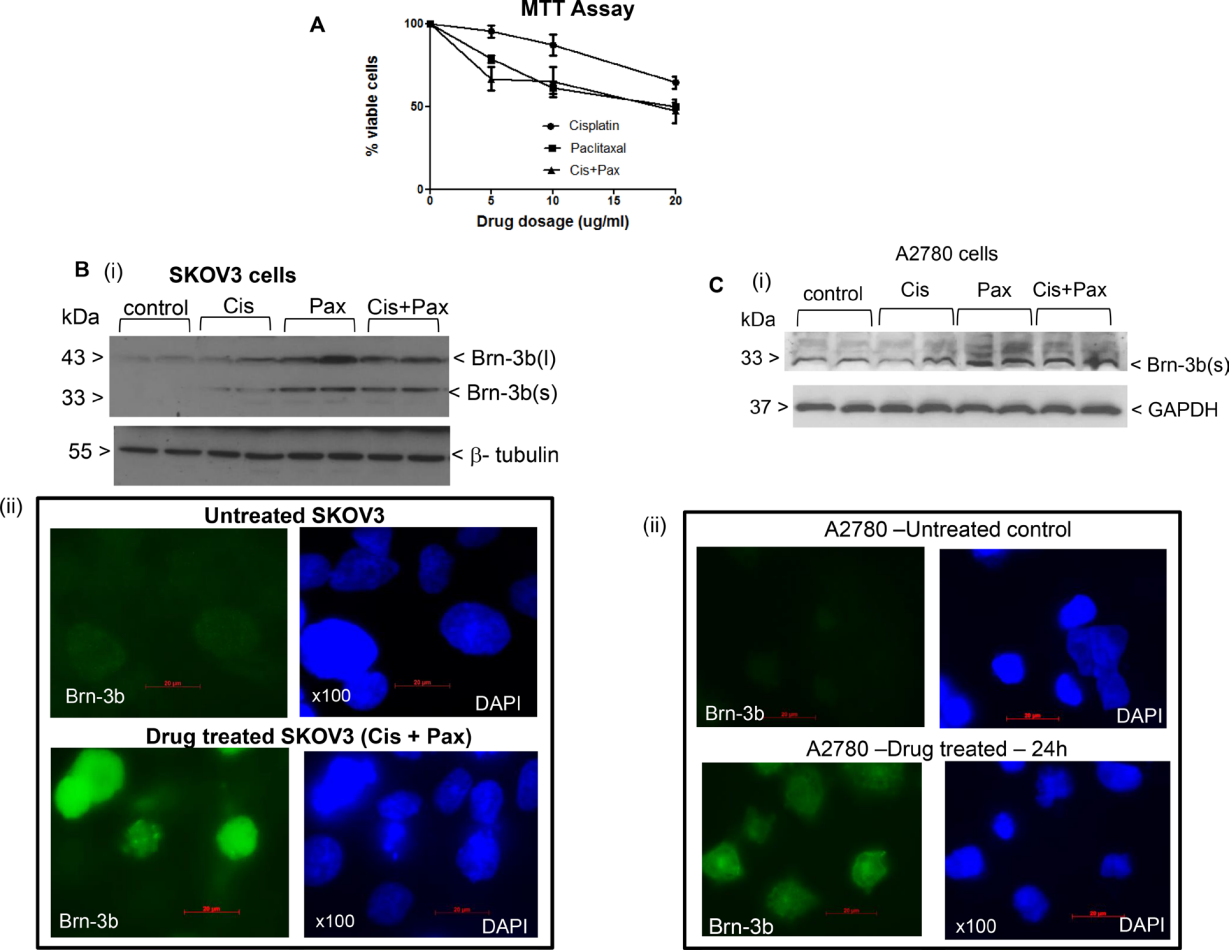
Brn-3b induction in drug treated SKOV3 cells (**A**) Results of MTT assays showing changes in viability of SKOV3 cells following treatment with different doses of cisplatin or paclitaxel (0–20 µg/ml) and combination of paclitaxel (1 µg/ml) and increasing cisplatin, as indicated. The percentage of viable cells following different treatments is expressed relative to control untreated cells, set at 100%. Values represent mean and standard error from three independent experiments each with at least three separate treatment sets. (**B**) (i) Representative western blots showing Brn-3b expression in untreated SKOV3 control cells and experimental cells treated with cisplatin (5 µg/ml) or paclitaxel (1 µg/ml) alone or combination of cis + pax (5 µg/ml +1 µg/ml). β-tubulin was used to control for protein loading. (ii) Representative immunofluorescence staining shows localisation of Brn-3b proteins in SKOV3 cells treated with drug combination (cisplatin + paclitaxel) for 24 h. Brn-3b protein was detected using FITC conjugated secondary Ab (green) and DAPI staining indicates the cell nuclei. (**C**) (i) Representative western blots showing Brn-3b expression in untreated A2780 control cells and experimental cells treated with cisplatin (5 µg/ml) or paclitaxel (5 µg/ml) alone or combination of cis + pax (5 µg/ml +1 µg/ml). GAPDH immunoblot was used to control for protein loading and was similar to another invariant protein, β-tubulin (not shown). (ii) Representative immunofluorescence staining shows localisation of Brn-3b proteins in A2780 cells treated with drug combination (cis + pax) for 24 h. Brn-3b protein was detected using FITC conjugated secondary Ab (green) and DAPI staining indicates the cell nuclei.

Since increased Brn-3b in neuroblastoma cells confers resistance to chemotherapeutic drugs such as cisplatin [11], which are commonly used with paclitaxel as first-line chemotherapeutic treatment of ovarian cancers, we were interested in studying Brn-3b expression in ovarian cancer cell lines following treatment with cisplatin and/or paclitaxel. The ovarian adenocarcinoma cell line, SKOV3 was used for preliminary studies in which MTT cell viability assays were undertaken to establish the optimal dose and time course for treatment (see Figure 1A). For individual drug treatment, increasing doses of cisplatin or paclitaxel were used as specified but for combination treatment, (cis+pax), 1 µg/ml paclitaxel was used while cisplatin was increased as specified. This was necessary to avoid high toxicity caused by increasing paclitaxel dosage. The results showed that while cisplatin treatment had a small effect on cell viability after 24 hours, increased doses of paclitaxel either alone or in combination caused reduced cell viability. Since combination therapies are more commonly used to treat ovarian cancers, all subsequent studies were undertaken using paclitaxel (1 µg/ml) + cisplatin (5 µg/ml) which caused consistent and reproducible cell loss (30–40%) by 24 hours.

To analyse for changes in Brn-3b expression following treatment, western blot analyses were undertaken using protein extracts prepared from SKOV3 cells that were either untreated (control) or treated as indicated. Figure 1B(i) shows a representative western blot demonstrating that Brn-3b was expressed at low levels in untreated cells but was increased following drug treatment for 24 hours. Treatment with paclitaxel or cis-pax combination caused significant increases in Brn-3b expression after 24 h when compared with untreated controls while cisplatin only treatment resulted in smaller changes. Two Brn-3b protein isoforms (l) and, (s) exist and while Brn-3b(l) was found at lower levels in untreated cells, both isoforms were increased following drug treatment. GAPDH antibodies and/ or β-tubulin levels were used to determine variation in protein loading between different samples.

Similar studies were carried out to analyse Brn-3b expression in protein extracts from untreated and drug treated A2780 cells, a high-grade serous ovarian cancer cell line. Figure 1C(i) shows a representative western blot which demonstrated lower Brn-3b protein expression in untreated control cells which was increased following drug treatment especially after cis + pax combination. However, protein levels appear to be lower than SKOV3 cells and only the shorter Brn-3b(s) protein was detected in these cells.

Immunofluorescence staining and fluorescent imaging were also carried out in SKOV3 and A2780 to confirm Brn-3b expression and to analyse for cellular localization in these cells with and without drug treatment. Representative images shown in Figure 1B (ii) demonstrates that low levels of Brn-3b protein in untreated SKOV3 was significantly increased following treatment with cis + pax (lower panel). As expected for a transcription factor, protein localisation appears to be mainly in the cell nuclei while lower levels in the cytoplasm may reflect newly synthesized proteins that have not yet been imported into the cell nuclei. Similar expression patterns were detected in A2780 [Figure 1C (ii)] confirming lower protein expression in untreated cells which is increased following drug treatment. Similar to SKOV3 cells, Brn-3b was primarily localised in the cell nuclei of drug treated A2780 cells, when compared with untreated controls.

### Brn-3b expression in drug resistant SKOV3 cells

**Figure 2:**
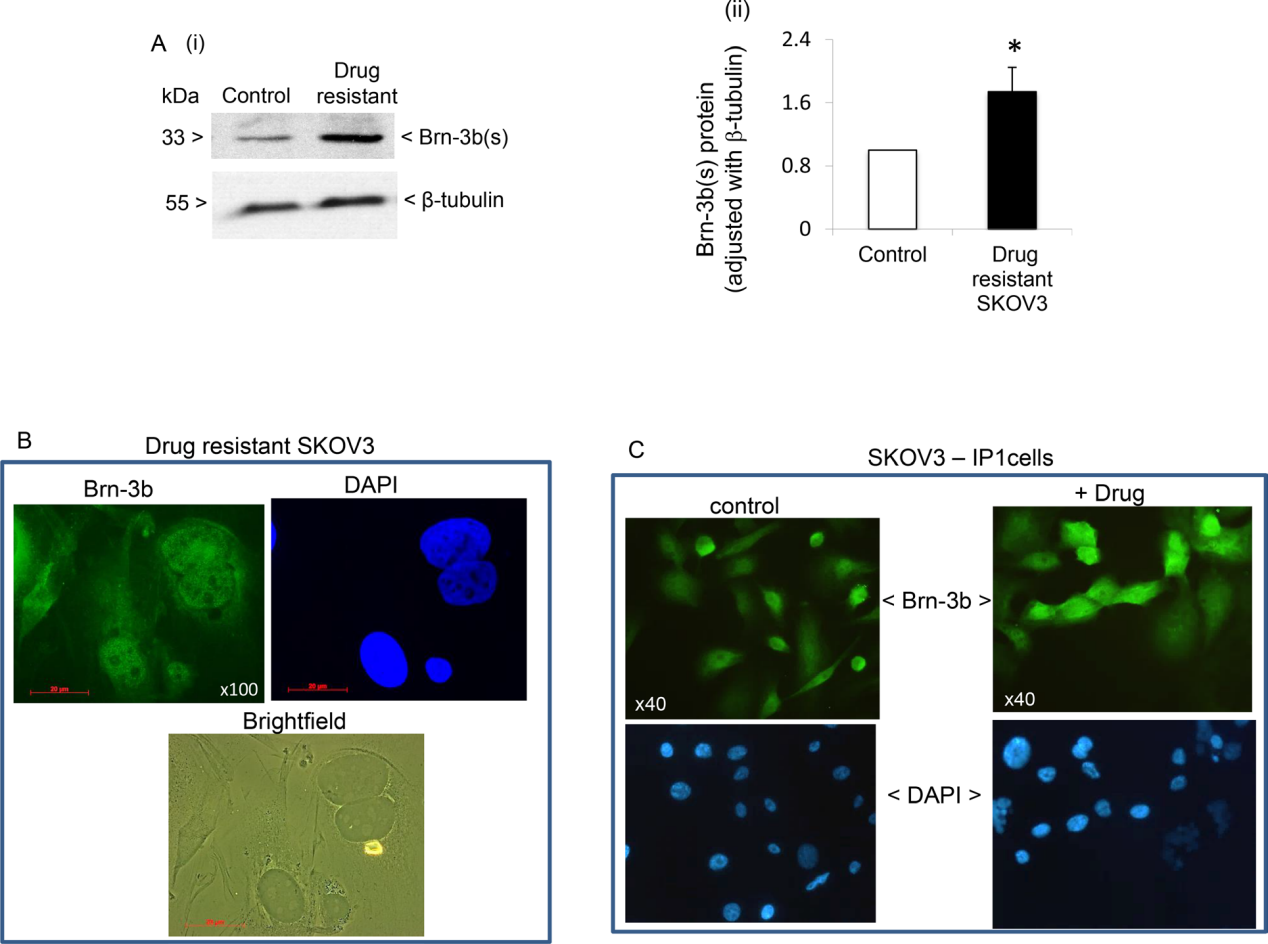
(**A**) (i) Representative western blot analysis showing increased Brn-3b protein expression in drug resistant cells compared with untreated control cells. (ii) Quantification of Brn-3b protein adjusted for β-tubulin in cisplatin resistant (drug-resistant) cells compared with control cells (set at 1). ^*^Indicates statistical increase in Brn-3b protein (*P <* 0.05, student’s *t* test). (**B**) Representative immunofluorescence staining for Brn-3b proteins in drug resistant SKOV3 cells (2 weeks). Brn-3b protein was detected using FITC conjugated secondary Ab (green) and DAPI staining indicates the cell nuclei. Phase contrast microscopy shows the cell morphology in bright field. (**C**) Representative immunofluorescence staining for Brn-3b proteins in metastatic SKOV3-IP1 either in untreated controls or following drug treatment with cis + pax. Brn-3b protein was detected using FITC conjugated secondary Ab (green) and DAPI staining indicates the cell nuclei.

Increased Brn-3b was previously linked to survival following drug treatment and invasiveness in other cancers [11]. Therefore, we next tested if Brn-3b expression was sustained in drug resistant SKOV3 cells which were grown under selection pressure for >2 weeks (see methods). Protein extracts from drug resistant cells were analysed for changes in Brn-3b expression using western blotting and a representative blot shown in Figure 2A (i) demonstrates that drug resistant cells expressed higher levels of Brn-3b protein when compared with untreated controls. Quantification of multiple experiments (*n* = 5) showed statistically significant increases in Brn-3b in drug resistant SKOV3 cells, when compared with untreated controls (*p* value < 0.05) [Figure 2A (ii)]. Figure 2B shows representative immunofluorescent staining of drug resistant cells grown on coverslips, which confirmed Brn-3b protein localisation in drug resistant cells (green). Bright field imaging displayed distinct morphological features of drug resistant cells including flattened cells with significant projections and large nuclei.

We also analysed Brn-3b expression in the metastatic IP1, which was derived from ascites of mice bearing SKOV3 tumours). Figure 2C shows a representative immunostaining image which demonstrated that Brn-3b was highly expressed in untreated cells but with increased intensity following drug treatment (cis + pax). When taken together with data from drug resistant cells, these results suggest that Brn-3b may be associated with drug resistance and metastasis in ovarian cancers also.

### Known Brn-3b target, HSP-27, closely correlates with Brn-3b expression

**Figure 3:**
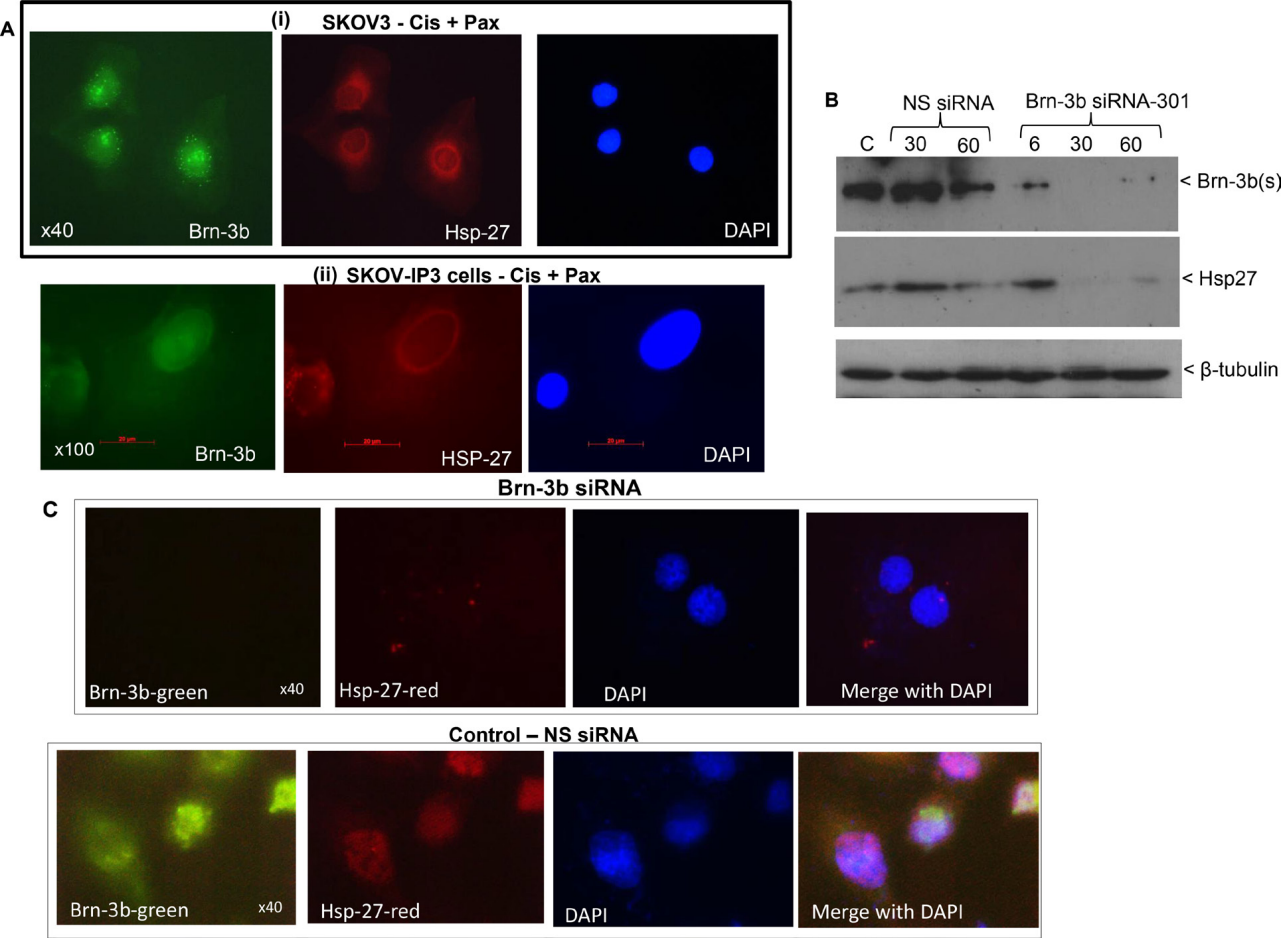
(**A**) Representative immunofluorescence staining for Brn-3b and HSP27 proteins in SKOV3 cells (i) or IP1 cells following drug treatment. Brn-3b protein was detected using FITC conjugated secondary Ab (green) and DAPI staining indicates the cell nuclei. (**B**) Representative western blot of SKOV3 cells transfected with Brn-3b siRNA-301 or non-silencing controls (NS siRNA) showing reduction of Brn3b protein levels and reduced HSP27 protein levels in cells transfected with Brn3b siRNA-301. β-tubulin blots were used to show variability in protein loading in different samples. (**C**) Representative immunofluorescence staining for Brn-3b and HSP27 proteins in SKOV3 cells transfected with Brn-3b siRNA-301 or non-silencing controls (NS siRNA) showing reduction of Brn3b protein levels and reduced HSP27 protein levels in cells transfected with Brn3b siRNA 301. Brn-3b protein was detected using FITC conjugated secondary Ab (green) whereas HSP27 Ab was detected using PE conjugated secondary Ab (red). DAPI staining indicates the cell nuclei. Images were captured at 40× magnification.

The small heat-shock protein, HSP27 is a known Brn-3b target gene [18] and in fact Brn-3b was shown to be required for maximal expression of HSP27 in drug treated breast cancer cells. Since HSP27 is strongly associated with aggressive ovarian cancers that has poor prognosis and reduced survival [20], we next tested if Brn-3b expression correlated with HSP27 expression in ovarian cancer cells also. Therefore, co-immunofluorescent staining was carried out in drug treated SKOV3 cells to analyse for co-expression of HSP27 and Brn-3b. Figure 3A shows that at the cellular level drug treated SKOV3 cells (i) or SKOV3 -IP3 cells (ii) which express nuclear Brn-3b (green) also expressed HSP27 (red) in the cytoplasm.

To determine if Brn-3b was required for HSP27 expression in these cells, we next tested if reducing Brn-3b expression in SKOV3 cells would also reduce HSP27 expression. Brn-3b reduction was achieved using MISSION^®^ short interfering RNA (siRNA) (Sigma, UK) designed to target Brn-3b protein and a non-silencing (NS) siRNA sequence was used as controls for these experiments. Different amounts (6, 30 and 60 pmol) of MISSION^®^ siRNA were transfected into SKOV3 cells and changes in Brn-3b protein expression were analysed by western blotting. Figure 3B shows that 30–60 pmol of Brn-3b SASI_HS01_00087301 siRNA (referred to as Brn-3b siRNA-301) could effectively and reproducibly reduce Brn-3b levels after 72 hours and as such 30 pmol was used for subsequent studies (see below). Importantly, cells transfected with Brn-3b siRNA expressed significantly reduced HSP27 protein compared with NS siRNA (middle panel). This was also confirmed by co-immunostaining studies since cells transfected with Brn-3bsiRNA-301 did not express HSP27 proteins whereas NS siRNA transfected cells clearly co-expressed Brn-3b and HSP27 (Figure 3C). These results suggest that Brn-3b may be involved in regulating HSP27 in ovarian cancer cells also and thereby contribute to drug resistance and metastatic potential in these cells.

### siRNA silencing of Brn-3b expression reduces cell viability in SKOV3 or IP1 cells

**Figure 4:**
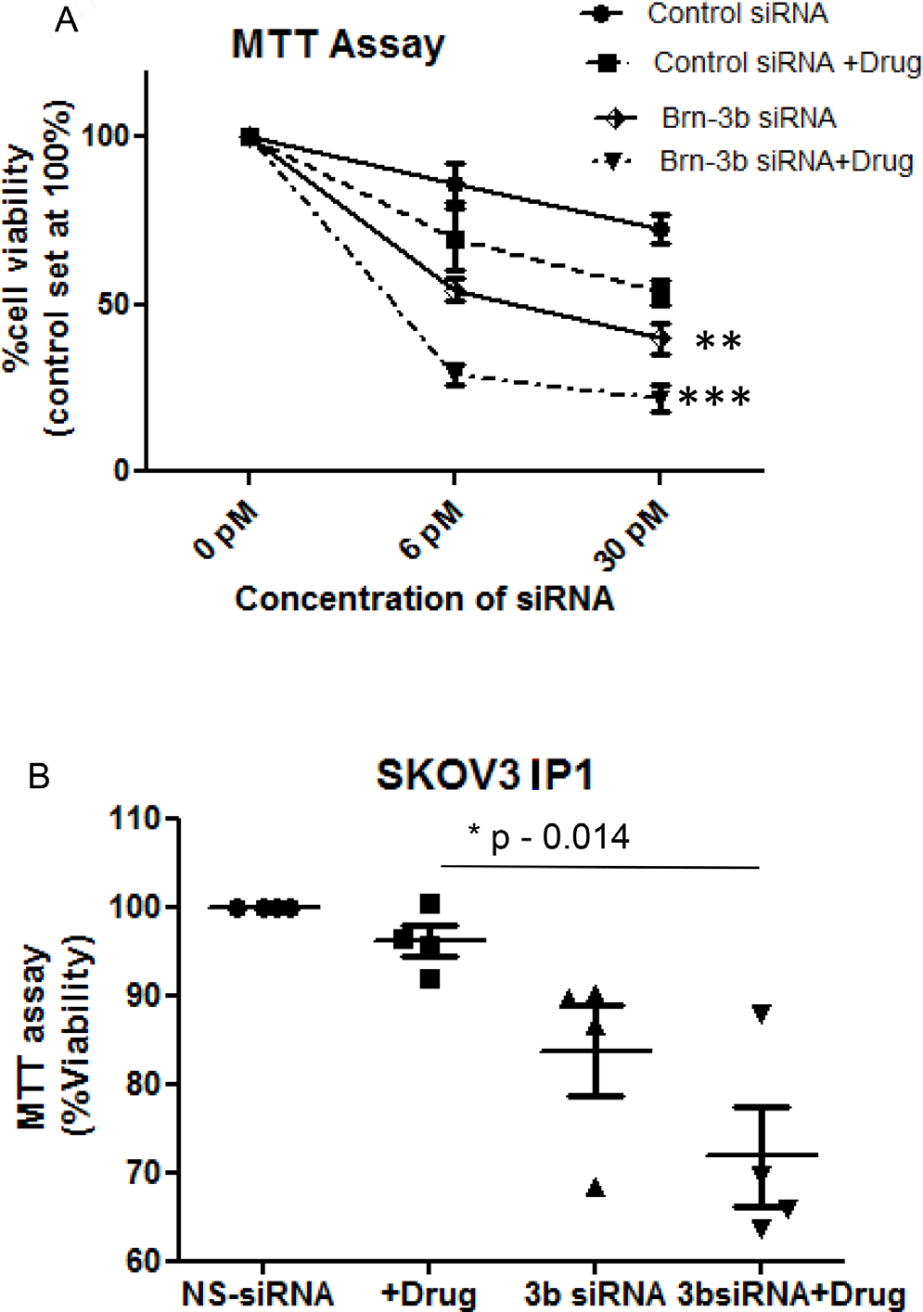
(**A**) Results of pooled MTT assays to analyse for changes in cell viability in SKOV3 cells transfected with Brn-3b siRNA-301 or non-silencing controls (NS siRNA) for 72 hours followed by treatment with cisplatin and paclitaxel (5 µg/ml +1 µg/ml). Results are expressed relative to untreated cells, where absorbance was set at 100 and changes in viability of cells transfected with NS siRNA or Brn-3b siRNA 301 are expressed relative to the control. Values represent the mean ± standard error from three independent experiments. (^***^P < 0.001, two-way ANOVA). (**B**) MTT assays to analyse for changes in cell viability in IP1 cells transfected with Brn-3b siRNA-301 for 72 hours followed by treatment with cisplatin and paclitaxel (5 µg/ml + 1 µg/ml). Results are expressed relative to untreated NS siRNA cells, where absorbance was set at 100.

To determine if Brn-3b was required for cell survival following drug treatment, we next tested if reducing Brn-3b expression affected cell fate. Therefore, SKOV3 cells were transfected with different concentrations of Brn-3b siRNA or NS siRNA (6 and 30 pmol) for 72 h. After 72 h, cells were either left untreated or treated with cis + pax combination for 24 h and cell viability was then analysed using MTT assay. Figure 4A shows percentage of viable cells under different conditions relative to untreated control, set at 100%. Non-silencing NS siRNA had minimal effects on cell viability in untreated cells but drug treatment caused an expected reduction in cell numbers. In contrast, Brn-3b siRNA-301 was sufficient to reduce cell numbers even in untreated cells (54% ± 9 at 6 pmol and 40% ± 13 at 30 pmol) when compared with the appropriate non-silencing control siRNA (86% ± 17 at 6 pmol and 75.3% ± 11 at 30 pmol) (*P* < 0.05). In addition, treatment with chemotherapeutic drugs caused significant loss of cells transfected with Brn-3b siRNA (28% ± 9 at 6pmol and 21% ± 10 at 30 pmol) (*P* < 0.001). These results strongly suggest that reducing Brn-3b was sufficient to prevent proliferation and/ or reduce viability of SKOV3 ovarian cancer cells alone but when reduction of Brn-3b was combined with drug treatment, this resulted in significant loss of cells when compared with non-silencing control cells treated with drugs.

Similar studies were undertaken using the aggressive and metastatic IP1 sub-line, which was derived from ascites of mice injected with SKOV3. As before, cells were transfected with non-silencing (NS) control siRNA or Brn-3b siRNA and then either left untreated or treated with drug combination (cis + pax) for 24 hours. Results of MTT assays showed that treatment with cis + pax had little effect on reducing viability of control IP1 cells (Figure 4B), suggesting drug resistance when compared with the parental SKOV3 cells. However, siRNA that targeted Brn-3b caused loss in cell viability even in untreated cells and more marked reduction in cell survival following drug treatment. Therefore similar to the SKOV3 cells, reducing Brn-3b increases sensitivity of a more aggressive, metastatic cell line to drug treatment.

## DISCUSSION

The acquisition of drug resistance by cancer cells is a major hurdle in treatment of patients with ovarian cancers since it underlies the recurrence of cancer cells that become intractable to treatment. This can profoundly affect outcome since it is associated with poor prognosis and reduced survival but the molecular mechanisms associated with such changes are not fully understood [9, 22]. The Brn-3b transcription factor is a master regulator that can control the expression of multiple target genes in a cell specific or growth condition dependent manner. It has been implicated in different tumourigenic processes in breast cancer and neuroblastoma cells since overexpression of this protein can enhance cell growth *in vitro* and tumour growth *in vivo* but also alter behaviour of the cells by increasing anchorage independent growth [10, 11]. Conversely, reducing Brn-3b was sufficient to inhibit cell proliferation *in vitro* and tumour growth *in vivo*. However, Brn-3b is also increased in cells treated with chemotherapeutic drugs such as cisplatin or doxorubicin and, under such conditions, Brn-3b can confer survival and resistance to treatment while increasing cell migration and metastatic potential [11, 12, 18].

Brn-3b has also been detected in ovarian cancer cells but its expression and effects in these cells have not been reported previously. In this study, we demonstrate that Brn-3b protein is increased in different ovarian cancer cells including SKOV3 and A2780, following treatment with common chemotherapeutic agents, cisplatin and paclitaxel. Analysis of SKOV3 cells shows that low Brn-3b levels in untreated cells is significantly increased in response to drug treatment particularly when using the combination of cisplatin and paclitaxel, commonly used for treatment of patients with ovarian cancers. The gene encoding Brn-3b can give rise to 2 protein isoforms, thought to arise from alternative promoter usage, which results in inclusion of an additional N-terminal domain in the longer Brn-3b(l) protein that is not present in the shorter Brn-3b(s) isoform [23, 24]. Interestingly, our data has shown distinct patterns of Brn-3b protein isoforms in ovarian cancer cells. For instance, the shorter isoform, Brn-3b(s) isoform is detected in both cell lines following drug treatment but appears to be the only isoform in A2780 cells. In contrast, the longer Brn-3b(l) isoform is detectable at low levels in untreated SKOV3 cells but also increases following drug treatment. Although the implications of such distinct expression patterns are still to be elucidated, previous studies have shown that Brn-3b(s) is increased in breast cancers or neuroblastoma tumours and its overexpression in breast cancer or neuroblastoma derived cell lines is sufficient to enhance growth and transformation [10, 11] but also confer resistance to chemotherapeutic drugs including cisplatin and doxorubicin [12]. As such, induction of the shorter Brn-3b (s) isoform following drug treatment of ovarian cancer cells may suggest that this protein will have similar effects in ovarian cancer cells also.

It is notable that, Brn-3b appears to be expressed at higher levels in SKOV3 cells, which are derived from metastatic cells in ascites taken from a patient with ovarian adenocarcinoma when compared with A2780, which was generated from primary ovarian endometrial adenocarcinoma. Since high levels of Brn-3b can confer drug resistance and migratory potential in breast cancer cells following treatment [18], we considered if this protein could have similar roles in controlling growth and behaviour of ovarian cancer cells also. In line with this, drug resistant SKOV3 cells, which were grown continuously in low doses of cis + pax treatment for >2 weeks, expressed high levels of Brn-3b, suggesting that this regulator may have potential roles in the acquisition of drug resistance in ovarian cancer cells. This is also supported by studies carried out in the highly metastatic SKOV3-IP1 sub line, which expressed Brn-3b at baseline but showed increased expression following drug treatment. Therefore, Brn-3b may regulate genes associated with drug resistance and migration, similar to its effects in other cancers [11, 18].

Previous studies have identified multiple Brn-3b target genes in cancer cells but whilst activation of target genes such as cyclin D1/CDK4 and repression of BRCA1 tumour suppressor are likely to contribute to growth and proliferation in cancer cells, the small heat shock protein, HSP27 was the main Brn-3b target gene, known to be associated with survival following drug treatment and increased metastasis. In this regard, Brn-3b is known to be a potent activator of HSP27 since it can directly activate the gene promoter but also co-operates with the oestrogen receptor (ER) to maximally stimulate HSP27 expression. Moreover, Brn-3b levels in biopsies taken from breast cancer patients also show strong correlation with elevated HSP27 expression. *In vitro* studies in breast cancer cell lines have also shown that Brn-3b is required for HSP27 expression in doxorubicin treated cells [12], since shRNA to silence Brn-3b was sufficient to block HSP27 expression. In this study, we demonstrate that in drug treated SKOV3 cells, high Brn-3b levels correlated well with induction of HSP27 protein. More importantly, siRNA to target Brn-3b resulted in loss of HSP27 protein confirming that Brn-3b may also regulate its expression in ovarian cancer cells.

In ovarian cancers, HSP27 is strongly implicated in survival, acquisition of drug resistance and metastatic potential in aggressive tumours [25]. Furthermore, knockdown of HSP-27 using siRNA has been shown to increase sensitivity to chemotherapeutic drugs such as paclitaxel in ovarian cancer cells [21], suggesting that expression of this heat shock protein is important for conferring survival effects in drug treated cells. Its pro-survival effects may arise from the ability of HSP27 to protect cells from apoptosis by inhibiting apoptosis through mitochondrial mediated caspase-3 dependent pathways or membrane (Fas) induced apoptosis [26]. In patients with epithelial ovarian cancers, increased serum expression of HSP27 correlated with peritoneal metastasis [19, 20] and elevated HSP27 is considered as a predictor of poor survival in patients with ovarian tumours. Therefore, high Brn-3b levels in ovarian cancers may cause increased expression of such target genes and thereby confer drug resistance and/or increased metastatic potential.

Finally, our data show that Brn-3b is required for survival of SKOV3 ovarian cancer cells because siRNA to reduce Brn-3b can result in loss of cell viability in untreated cells but more importantly, reducing Brn-3b appears to confer increased sensitivity to chemotherapeutic drugs. Similarly, Brn-3b is expressed at high levels in the aggressive, metastatic IP1 sub-line, derived from ascites in mice injected with SKOV3 cells. Drug treatment of IP1 cells targeted with the non-silencing siRNA had little effect on cell viability suggesting that these cells had increased resistance to treatment compared with the parental SKOV3 cells, reducing Brn-3b using siRNA was sufficient to reduce cell viability but also increased sensitivity to drug treatment. These findings may be highly relevant to drug resistant cancers because whilst many patients show robust responsiveness to early chemotherapeutic treatment, the majority of patients develop relapsed drug resistant cancer which become refractory to subsequent treatment [8].

This is the first report showing that increased Brn-3b transcription factor may be important for controlling growth and behaviour of human ovarian cancer cells, particularly following treatment with common chemotherapeutic agents such as cisplatin and paclitaxel. These results, when combined with data from studies in other cancer related models [10–14, 18, 27], points to an important role for Brn-3b in promoting survival and drug resistance in ovarian cancer cells. Based on its known function, the effects of Brn-3b and growth and behaviour of cancer cells are likely to be mediated by its ability to regulate the expression of multiple target genes that drive specific cellular effects. However whilst its growth promoting effects are linked to transactivation of cell cycle target genes such as cyclin D1 [14, 15] and repression of tumour suppressor genes such as BRCA1 [13], its effects in drug treated cells are likely to be driven by activation of the small heat-shock protein, HSP27 [18] which can confer protection against apoptosis and accumulation of DNA damage e.g. caused by reactive oxygen species (ROS) [26, 28]. Its effects on diverse target genes may explain why reducing Brn-3b can sensitize cells to further chemotherapeutic treatment.

Although Brn-3b expression in different ovarian cancers and its mechanism of action in initiation and progression of this disease are still to be elucidated, our results suggest that increased expression of this regulator can contribute to survival and drug resistance in ovarian cancer cells following chemotherapy. As such, Brn-3b may provide a novel but important therapeutic target that for the treatment of recurrent drug-resistant ovarian cancers, which contribute to the deadly nature of this disease.

## MATERIALS AND METHODS

### Cell culture

Human SKOV3 ovarian adenocarcinoma cells (ATCC HTB-77^™^) were grown in full growth medium [McCoy’s 5A medium with 10% foetal bovine serum + 1% penicillin-streptomycin (Gibco, Invitrogen)]. Human SKOV3-IP1cells were derived from the ascetic fluid of mice bearing SKOV3 tumours and for clarity will be referred to as IP1 cells. Cells were grown in full growth medium [RPMI medium with 10% foetal bovine serum + 1% penicillin-streptomycin (Gibco, Invitrogen)]. Human A2780 ovarian (epithelial) carcinoma cells (Sigma, 93112519) were also cultured in full growth medium [RPMI medium with 10% foetal bovine serum + 1% penicillin-streptomycin (Gibco, Invitrogen)]. SKOV3 iP1 and A2780 cells were provided by Dr. Timothy Witney, University College London. Cells were maintained in a humidified atmosphere at 37**°** C and 5% CO_2_ and sub-cultured upon reaching 70–80% confluence. For experiments, cells were plated in 6-well (1–5 × 10^5^ cells /well) or 12-well cell culture plates (1–5 × 10^4^ cells/well) prior to other protocols/treatments. Dose-response studies were used to establish the effects of different amounts of chemotherapeutic drugs, either alone or together, on cell viability and gene expression. For studies with the drug combinations, 1 µg/ml of paclitaxel was used with increasing amounts of cisplatin because at higher doses, paclitaxel in combination with cisplatin resulted in more significant cell death than similar doses alone. For generating drug-resistant cells, SKOV3 cells were treated with 1 µg/ml paclitaxel and 5 µg/ml cisplatin for 24 hours to kill off the most sensitive cells after which cells were maintained in medium containing 1 µg/ml of cis + 0.1 µg/ml pax. Medium with fresh drug was changed every 2–3 days for the duration of up to or > 2 weeks.

### Transfection with short interfering RNA (siRNA) to reduce gene expression

Different pre-designed MISSION^®^ siRNAs (Sigma, UK) were tested for effects on reducing Brn-3b expression in SKOV3 cells by transfecting different amounts of siRNA (6, 30 and 60 pmol), using the MISSION^®^ siRNA Transfection Reagent, in accordance with the manufacturer’s protocol. This was compared with cells transfected with an unrelated non-silencing siRNA that was used as a control. Western blot analysis of total cellular proteins taken at different times (72–96 hours) after transfection was used to determine effectiveness of knockdown. In addition, to study the effects of chemotherapeutic drugs after reduction of Brn-3b, transfected cells were treated with different drug combinations for 24 hours before further analysis (e.g. MTT assays).

### Protein extraction and immunoblotting

Proteins harvested from SKOV3, IP1 and A2780 cells (in 2X Laemmli buffer) were resolved by 12% SDS-PAGE and used for immunoblotting as described (7). Briefly, membranes were blocked for 1 h in phosphate-buffered saline containing 0.1% Tween 20 (PBST)/4% non-fat powdered milk. Primary antibody (1:1000 dilution) was incubated overnight at 4° C or for 2–3 h at room temperature (RT). Following 5x washes with PBST, secondary antibody (1:2000) was incubated for 1 h (RT). Blots were developed using enhanced chemiluminescence reagent (Pearce, UK). Differences in total protein levels were adjusted using housekeeping genes, e.g. β-tubulin or GAPDH.

### MTT assays for cell viability

MTT assays were used to assess changes in live cells by determining the rate at which viable cells can reduce the tetrazolium dye [3-(4, 5 dimethylthizol-2-yl) 2–5 diphenyltetrazolium bromide] to its insoluble formazan salt. These assays were undertaken using standard protocols whereby treated cells and untreated controls (SKOV3, IP1 and A2780 cell lines) were incubated with 250 μl of 0.5 mg/ml of MTT for 2 hrs at 37° C. Following aspiration of MTT, formazan crystals were dissolved in 200 μl DMSO and optical density was measured by absorbance spectrometry at 560 nm.

### Immunofluorescence

SKOV3, SKOV3 iP1 and A2780 cells were grown on coverslips and treated with cis+pax for 24 h. Cells were fixed in 4% paraformaldehyde (PFA) for 15 min, washed in 1X Phosphate-buffered saline (PBS) and pre-incubated in block solution [20% goat serum in PBS + 0.1% triton-X100 (PBST)] for 30–60 min before incubation with primary Brn-3b antibody (1:500) (overnight at 4° C). Following 5 washes in PBST, secondary antibody was incubated (1 hr, RT). After final washes, cells were mounted in fluorescent mounting medium with DAPI (Vector Laboratories). Images were captured using the ZEISS Axioskop microscope with Axiovision software (Zeiss).

### Statistical analysis

Data was analysed using Graphpad Prism with the student’s *t*-test use for normal distribution or the Man–Whitney *U* test where data was not normally distributed. Two-way analysis of variance (ANOVA) and post-hoc test (e.g. bonferroi) were used to determine whether the differences in the means of two or more factors were significant. All data in text and figures are presented as means ± SD with *P* < 0.05 indicating statistical significance (^*^P < 0.05).

## Abbreviations

Brn-3b: (POU4F2 Brn-3b)
Pax: (paclitaxel)
Cis: (cisplatin)
siRNA: (Short interfering RNA)
MTT assay: (3-(4,5-dimethylthiazol-2-yl)-2,5-diphenyltetrazolium bromide)

## References

[1] Sankaranarayanan R, Ferlay J (2006). Worldwide burden of gynaecological cancer: the size of the problem. Best Pract Res Clin Obstet Gynaecol.

[2] Cho KR, Shih IM (2009). Ovarian cancer. Annu Rev Pathol.

[3] Bast RC, Hennessy B, Mills GB, Mills GB (2009). The biology of ovarian cancer: new opportunities for translation. Nat Rev Cancer.

[4] Vaughan S, Coward JI, Bast RC, Berchuck A, Berek JS, Brenton JD, Coukos G, Crum CC, Drapkin R, Etemadmoghadam D, Friedlander M, Gabra H, Kaye SB (2011). Rethinking ovarian cancer: recommendations for improving outcomes. Nat Rev Cancer.

[5] McGuire WP, Hoskins WJ, Brady MF, Kucera PR, Partridge EE, Look KY, Clarke-Pearson DL, Davidson M (1997). Comparison of combination therapy with paclitaxel and cisplatin versus cyclophosphamide and cisplatin in patients with suboptimal stage III and stage IV ovarian cancer: a Gynecologic Oncology Group study. Semin Oncol.

[6] Piccart MJ, Bertelsen K, James K, Cassidy J, Mangioni C, Simonsen E, Stuart G, Kaye S, Vergote I, Blom R, Grimshaw R, Atkinson RJ, Swenerton KD (2000). Randomized intergroup trial of cisplatin-paclitaxel versus cisplatin-cyclophosphamide in women with advanced epithelial ovarian cancer: three-year results. J Natl Cancer Inst.

[7] Colombo N, Parma G, Bocciolone L, Sideri M, Franchi D, Maggioni A (1999). Role of chemotherapy in relapsed ovarian cancer. Crit Rev Oncol Hematol.

[8] Torri V, Harper PG, Colombo N, Sandercock J, Parmar MK (2000). Paclitaxel and cisplatin in ovarian cancer. J Clin Oncol.

[9] Agarwal R, Kaye SB (2003). Ovarian cancer: strategies for overcoming resistance to chemotherapy. Nat Rev Cancer.

[10] Dennis JH, Budhram-Mahadeo V, Latchman DS (2001). The Brn-3b POU family transcription factor regulates the cellular growth, proliferation, and anchorage dependence of MCF7 human breast cancer cells. Oncogene.

[11] Irshad S, Pedley RB, Anderson J, Latchman DS, Budhram-Mahadeo V (2004). The Brn-3b transcription factor regulates the growth, behavior, and invasiveness of human neuroblastoma cells *in vitro* and *in vivo*. J Biol Chem.

[12] Fujita R, Ounzain S, Wang AC, Heads RJ, Budhram-Mahadeo VS (2011). Hsp-27 induction requires POU4F2/Brn-3b TF in doxorubicin-treated breast cancer cells, whereas phosphorylation alters its cellular localisation following drug treatment. Cell Stress Chaperones.

[13] Budhram-Mahadeo V, Ndisang D, Ward T, Weber BL, Latchman DS (1999). The Brn-3b POU family transcription factor represses expression of the BRCA-1 anti-oncogene in breast cancer cells. Oncogene.

[14] Budhram-Mahadeo VS, Irshad S, Bowen S, Lee SA, Samady L, Tonini GP, Latchman DS (2008). Proliferation-associated Brn-3b transcription factor can activate cyclin D1 expression in neuroblastoma and breast cancer cells. Oncogene.

[15] Samady L, Dennis J, Budhram-Mahadeo V, Latchman DS (2004). Activation of CDK4 gene expression in human breast cancer cells by the Brn-3b POU family transcription factor. Cancer Biol Ther.

[16] Samady L, Faulkes DJ, Budhram-Mahadeo V, Ndisang D, Potter E, Brabant G, Latchman DS (2006). The Brn-3b POU family transcription factor represses plakoglobin gene expression in human breast cancer cells. Int J Cancer.

[17] Alaee M, Danesh G, Pasdar M (2016). Plakoglobin Reduces the *in vitro* Growth, Migration and Invas ion of Ovarian Cancer Cells Expressing N-Cadherin and Mutant p53. PLoS One.

[18] Lee SA, Ndisang D, Patel C, Dennis JH, Faulkes DJ, D’Arrigo C, Samady L, Farooqui-Kabir S, Heads RJ, Latchman DS, Budhram-Mahadeo VS (2005). Expression of the Brn-3b transcription factor correlates with expression of HSP-27 in breast cancer biopsies and is required for maximal activation of the HSP-27 promoter. Cancer Res.

[19] Zhao M, Ding JX, Zeng K, Zhao J, Shen F, Yin YX, Chen Q (2014). Heat shock protein 27: a potential biomarker of peritoneal metastasis in epithelial ovarian cancer?. Tumour Biol.

[20] Langdon SP, Rabiasz GJ, Hirst GL, King RJ, Hawkins RA, Smyth JF, Miller WR (1995). Expression of the heat shock protein HSP27 in human ovarian cancer. Clin Cancer Res.

[21] Song TF, Zhang ZF, Liu L, Yang T, Jiang J, Li P (2009). Small interfering RNA-mediated silencing of heat shock protein 27 (HSP27) Increa ses chemosensitivity to paclitaxel by increasing production of reactive oxygen species in human ovarian cancer cells (HO8910). J Int Med Res.

[22] Piccart MJ, Lamb H, Vermorken JB (2001). Current and future potential roles of the platinum drugs in the treatment of ovarian cancer. Ann Oncol.

[23] Martin SE, Mu X, Klein WH (2005). Identification of an N-terminal transcriptional activation domain within Brn3b/POU4f2. Differentiation.

[24] Liu YZ, Dawson SJ, Latchman DS (1996). Alternative splicing of the Brn-3a and Brn-3b transcription factor RNAs is regulated in neuronal cells. J Mol Neurosci.

[25] Arts HJ, Hollema H, Lemstra W, Willemse PH, De Vries EG, Kampinga HH, Van der Zee AG (1999). Heat-shock-protein-27 (hsp27) expression in ovarian carcinoma: relation in response to chemotherapy and prognosis. Int J Cancer.

[26] Budhram-Mahadeo VS, Heads RJ, Calderwood SK, Sherman MY, Ciocca DR (2007). Heat Shock Protein-27 (Hsp-27) in Breast Cancers: Regulation of Expression and Function. Heat Shock Proteins in Cancer. Heat Shock Proteins, vol 2.

[27] Ounzain S, Bowen S, Patel C, Fujita R, Heads RJ, Budhram-Mahadeo VS (2011). Proliferation-associated POU4F2/Brn-3b transcription factor expression is regulated by oestrogen through ERα and growth factors via MAPK pathway. Breast Cancer Res.

[28] Saunders JA, Rogers LC, Klomsiri C, Poole LB, Daniel LW (2010). Reactive oxygen species mediate lysophosphatidic acid induced signaling in ovarian cancer cells. Free Radic Biol Med.

